# A comparative study of survival models for breast cancer prognostication revisited: the benefits of multi-gene models

**DOI:** 10.1186/s12859-018-2430-9

**Published:** 2018-11-03

**Authors:** Michal R. Grzadkowski, Dorota H. Sendorek, Christine P’ng, Vincent Huang, Paul C. Boutros

**Affiliations:** 1 Ontario Institute for Cancer Research, Toronto, Canada; 2 Department of Medical Biophysics, University of Toronto, Toronto, Canada; 3 Department of Pharmacology & Toxicology, University of Toronto, Toronto, Canada

**Keywords:** Multi-gene models, Single-gene models, Survival models

## Abstract

**Background:**

The development of clinical -omic biomarkers for predicting patient prognosis has mostly focused on multi-gene models. However, several studies have described significant weaknesses of multi-gene biomarkers. Indeed, some high-profile reports have even indicated that multi-gene biomarkers fail to consistently outperform simple single-gene ones. Given the continual improvements in -omics technologies and the availability of larger, better-powered datasets, we revisited this “single-gene hypothesis” using new techniques and datasets.

**Results:**

By deeply sampling the population of available gene sets, we compare the intrinsic properties of single-gene biomarkers to multi-gene biomarkers in twelve different partitions of a large breast cancer meta-dataset. We show that simple multi-gene models consistently outperformed single-gene biomarkers in all twelve partitions. We found 270 multi-gene biomarkers (one per ~11,111 sampled) that always made better predictions than the best single-gene model.

**Conclusions:**

The single-gene hypothesis for breast cancer does not appear to retain its validity in the face of improved statistical models, lower-noise genomic technology and better-powered patient cohorts. These results highlight that it is critical to revisit older hypotheses in the light of newer techniques and datasets.

**Electronic supplementary material:**

The online version of this article (10.1186/s12859-018-2430-9) contains supplementary material, which is available to authorized users.

## Background

The abundance of cheap and accurate genomic technologies has led to a multitude of different approaches for classifying breast cancer patients into different prognostic groups based on their transcriptomic profiles. These risk classifications are clinically useful because they allow the targeting of aggressive treatment on patients most vulnerable to tumour recurrence or mortality while avoiding exposing those with lower risk to associated side-effects. Prognostic signatures, also referred to as biomarkers, are a popular class of tools for converting mRNA abundance data into patient risk scores that can serve as proxies for clinical outcome. Several particularly proficient biomarkers for breast cancer have already proven to be commercially viable [1–3].

A biomarker generally consists of two parts: a gene set chosen for association with prognosis using a supervised or unsupervised feature selection algorithm and a risk score model that transforms the mRNA abundance levels from these genes into risk scores for a given patient cohort. Many biomarkers developed for breast cancer rely on complex algorithms for feature selection and risk score calculation [4–9]. However, it has been demonstrated that gene sets selected for prognostic ability using such methods often fail to outperform randomly chosen gene sets of the same size [10, 11]. This is concordant with a previous finding that large numbers of non-overlapping gene sets are associated with breast cancer prognosis [12, 13]. This appears to be a general characteristic of multiple tumour types [14, 15]. If the background or “null” level of gene set prognostic performance is relatively high, it makes it more challenging for feature selection algorithms to identify groups of genes that perform significantly better than random chance. It is therefore apparent that the fundamental properties of multi-gene biomarkers must be fully elucidated in order to identify optimal biomarkers, which is difficult to do even when the feature-size is pre-set.

Haibe-Kains et al. [16] cast further doubt on the usefulness of multi-gene biomarkers for breast cancer outcome prognosis by suggesting that they do not consistently outperform the simplest possible model: one based on a single, well-chosen gene. They found that a simple biomarker that dichotomizes patients based on the expression of the gene AURKA fared roughly as well as much more complex methods that attempt to leverage the mRNA abundance data of many genes or even the whole genome. Haibe-Kains et al. [16] thus put forward a “single-gene hypothesis”: there is little marginal utility in implementing multi-gene prognostic biomarkers with complex feature selection and patient risk scoring components in breast cancer. Given the other difficulties inherent in using multi-gene biomarkers outlined above, it would seem that single-gene biomarkers based on biological intuition confer an advantage in interpretability and ease of discovery without compromising prognostic performance.

However, in the decade since the single-gene hypothesis was formulated, there have been many advances in both genomics technology itself and in the analytical techniques used for biomarker development. The drop in cost of measuring mRNA abundance has led to the availability of a much greater number of datasets. One of the largest of these is the Metabric dataset, comprising nearly two thousand patients [17]. The improved fidelity of transciptomics technologies has also led to smaller levels of noise in newer expression datasets. Furthermore, bioinformaticians are continually finding new and improved ways of applying insights from the field of machine learning to biomarker development.

Given these new developments, we revisited the single-gene hypothesis, testing it on a meta-dataset of 4960 breast cancer patient expression profiles. We tested the prognostic performance of single-gene models, including AURKA, and two different types of multi-gene models on a deep sample of the biomarker population. This approach allowed us to draw generalizable insights into the relative performance of multi-gene and single-gene transcriptomic biomarkers for predicting breast cancer patient prognosis.

## Methods

**Fig. 1:**
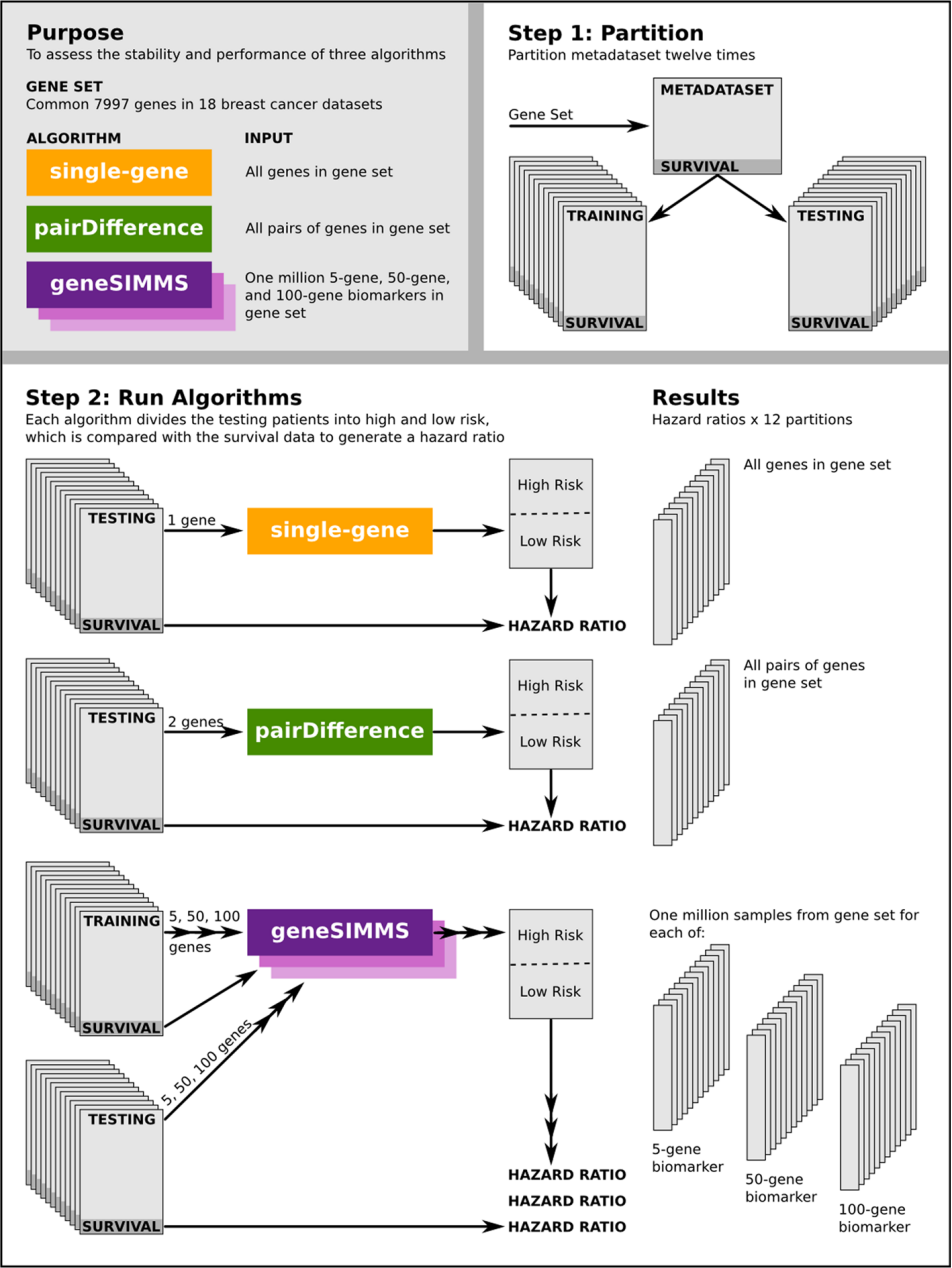
A summary of the experiment design

Figure 1 is a schematic outline of the analysis in which we comprehensively tested the stability and prognostic ability of single-, paired- and multi-gene biomarkers sampled from a pool of 7997 genes on various partitions of a 4960 patient gene expression meta-dataset. All computations were performed in the R statistical environment (v3.0.1) except for dataset pre-processing which was performed in R v2.13.0. All visualizations were created using the lattice (v0.20-15), latticeExtra (v0.6-24) and RColorBrewer (v1.0-5) packages, except for Fig. 1 which was created using Inkscape v0.48.

### Meta-dataset preparation and partition

We collected eighteen publicly-available raw breast cancer mRNA abundance datasets for which patient survival data was included (Table 1). Training and testing sample cohorts obtained from the same study were treated as separate datasets. Pre-processing and normalization techniques were applied independently to each of the eighteen datasets, including those originating from the same chip type. For all except the two Metabric datasets, mRNA abundance levels were normalized using the RMA algorithm [18], as implemented in the R package affy (v1.28.0). Probes were mapped to Entrez IDs using custom CDFs (R packages hgu133ahsentrezgcdf v12.1.0, hgu133bhsentrezgcdf v12.1.0, hgu133plus2hsentrezgcdf v12.1.0, hthgu133ahsentrezgcdf v12.1.0 and hgu95av2hsentrezgcdf v12.1.0) [19]. For the Metabric datasets, pre-processing, summarization and quantile-normalization was performed on raw expression files generated by Illumina BeadStudio (R packages beadarray v2.4.2 and illuminaHuman v3.db-1.12.2). Any genes that did not have mRNA abundance measurements in all eighteen datasets were removed from the study. This resulted in a single meta-dataset of 7997 genes and 4960 patients.

**Table 1:** The eighteen breast cancer datasets used in this study, with the total number of unique patients and the microarray platform used in each

Study	PubMed ID	Patient Count	Platform
Bild	16273092	158	HG-U95AV2
Chin	17157792	129	HTHG-U133A
Desmedt-1	17545524	198	HG-U133A
Desmedt-2	21422418	107	HG-U133-PLUS2
Hatzis-1	21558518	310	HG-U133A
Hatzis-2	21558518	198	HG-U133A
Ivshina	17079448	249	HG-U133A/B
Metabric-Training	22522925	996	HumanHT-12-v3
Metabric-Validation	22522925	992	HumanHT-12-v3
Miller	16141321	236	HG-U133A/B
Pawitan	16280042	159	HG-U133A/B
Sabatier	20490655	252	HG-U133-PLUS2
Schmidt	18593943	200	HG-U133A
Sotiriou	16478745	94	HG-U133A
Symmans-JBI	20697068	65	HG-U133A
Symmans-MDA	20697068	195	HG-U133A
Wang	15721472	286	HG-U133A
Zhang	18821012	136	HG-U133A

We created twelve balanced partitions of this meta-dataset, each partition dividing the total patient cohort into a training set and a testing set such that neither cohort contained fewer than 46% (i.e. 2,282/4,960) of the total number of patients (Additional file 1: Table S1). All possible partitions meeting this criteria were identified and twelve partitions were chosen at random. Note that partitioning was done without splitting individual datasets between training and testing cohorts so that each dataset was entirely within one cohort or the other.

### Outcome prognosis models

Let *P* be a set of patients and let *G* be a set of genes, with *e*_*p*,*g*_ denoting the mRNA abundance level of gene *g*∈*G* in patient *p*∈*P*. The goal of a prognostic biomarker is to divide *P* into a low-risk group *P*_*l*_ and a high-risk group *P*_*h*_ such that the difference in survival between the two groups is maximized. Biomarkers consist of a subset of *G* used to assign patients to one of the two risk groups. To identify the most successful biomarkers we consider the hazard ratio. This is calculated by fitting a univariate Cox proportional hazards model (R package survival v2.37-4) to the survival data of the *P*_*l*_ and *P*_*h*_ patients.

#### single-gene

The single-gene model uses one gene *g*∗ as *G*. Patients are ranked in descending order of *e*_*p*,*g*∗_ and then split at the median expression level to produce *P*_*h*_ and *P*_*l*_. This is analogous to the AURKA gene model described by Haibe-Kains et al. [16].

#### pairDifference

The pairDifference model uses two genes (*g*_1_,*g*_2_), with patients divided according to the rule:

1 $$ p \in \left\{\begin{array}{ll} P_{h}, &\ \text{if}\ e_{p,g_{2}}\!\!>\!e_{p,g_{1}} \\ P_{l}, &\ \text{otherwise} \end{array}\right.  $$

PairDifference is a rank-based method that classifies samples by the mRNA abundance score ratios of gene pairs, creating a simple multi-gene model that remains easily biologically interpretable. This method is based on the well-studied top scoring pairs classifier which was previously tested on breast cancer expression data [20, 21].

#### geneSIMMS

As a prototypical multi-gene model, we used the *geneSIMMS* approach, which can incorporate a set of genes *G*^∗^ of arbitrary size. It also requires an independent training patient cohort *P*^*Tr*^. GeneSIMMS takes the normalized mRNA abundance levels of each gene for all patients (training and testing cohorts combined) and scales them to z-scores. For each *g*∈*G*^∗^, it then median dichotomizes *P*^*Tr*^ by the transformed mRNA abundance score and fits a univariate Cox proportional hazards model using only the training cohort’s survival information to get a hazard ratio HR _*g*_. A risk score is calculated for each combination of patient and gene using the formula:

2 $$ \text{risk}_{p,g} = \log_{2}(\text{HR}_{g}) \times \mathrm{e}_{p,g}  $$

A multivariate Cox proportional hazards model is then fit using these risk scores and training cohort survival data to get a Cox beta *β*_*g*_ for each *g*∈*G*^∗^. These betas are used to calculate risk scores for each patient *p*∈*P*^*Tr*^∪*P* using the formula:

3 $$ \text{risk}_{p} = \sum\limits_{g \in G^{\ast}} \left(\beta_{g} \times \text{risk}_{p,g}\right)  $$

The testing cohort patients are then dichotomized into *P*_*l*_ and *P*_*h*_ using the median of the risk scores calculated from the training cohort patients. geneSIMMS is implemented in the R package SIMMS v(0.0.1) and is easily scaled to a sub-network approach, as outlined elsewhere by Haider et al. [9], although this aspect is not evaluated in the present study to retain the focus on the single-gene hypothesis.

### Choosing and testing biomarkers

To thoroughly assess the performance of the three model types under consideration, we tested as many sets of genes as possible with each. Since the single-gene and pairDifference approaches are computationally inexpensive, we were able to test all possible gene sets for both of these algorithms: i.e. 7997 single-gene biomarkers and 31,872,006 two-gene biomarkers. Due to the massive population of possible gene sets and the computational complexity associated with geneSIMMS, we were unable to test all possible gene combinations and instead selected a random subset of gene sets to analyze. We thus sampled, without replacement, a million gene sets of sizes 5, 50 and 100 from the set of 7997 common genes for a total of 3,000,000 unique geneSIMMS biomarkers. For the purposes of exploring the effect of gene set size on biomarker performance we considered these three sets separately in our analysis, labelling them geneSIMMS-5, geneSIMMS-50, and geneSIMMS-100.

For each model, the corresponding biomarkers were tested on each of the twelve partitions described above. For single-gene and pairDifference, only the testing cohorts were used as these models do not require training data. This resulted in twelve matching performance measurements for each gene set for a given model.

### Comparison of single-gene and multi-gene methods

To assess the performance of methods yielding single gene biomarkers against those yielding multi-gene biomarkers, we compared AURKA to geneSIMMS on random sets of five genes. To ensure a fair comparison, rather than testing millions of gene sets, we restricted the number of random five gene sets to the number of genes evaluated by AURKA across 12 partitions. For the AURKA method, the cut point was determined using training set and performance was evaluated on the test set. Similarly, geneSIMMS was performed as described above but with some alterations. Specifically, z-scoring was performed independently on each cohort of data before being combined into their respective training and test sets, and to prevent information leakage, log2(HR_*g*_) and *β*_*g*_ were calculated using only the training set and used to determine patient risk scores for the test set. We labelled this set of geneSIMMS models geneSIMMS5-R.

The best performing biomarker in terms of |*log*_2_HR| was retrieved from each partition for both AURKA and geneSIMMS5-R. A two-tailed, homoscedastic paired T-test was used to determine if the two sets of hazard ratios are significantly different.

### Measuring biomarker performance stability

To compare the concordance of biomarker performance between the twelve meta-dataset partitions tested, we used the Concordance Correlation Coefficient (CCC) as introduced by Lin (1989) and further amended in Lin (2000). Biomarker performance was defined by the unadjusted hazard ratio returned by a univariate Cox proportional hazards model as described above. The formula for CCC for a given set of biomarkers and *n* meta-dataset partitions is:

4 $$ \frac{2 \! {\sum\nolimits}_{i=1}^{n} \! {\sum\nolimits}_{j=1}^{n}\sigma_{i,j}}{{\sum\nolimits}_{i=1}^{n} \! {\sum\nolimits}_{j=1}^{n} \! \left(\frac{\left(\mu_{i} - \mu_{j}\right)^{2}}{2}\right) + \left(n-1\right) \! {\sum\nolimits}_{i=1}^{n}\sigma_{i}^{2}}  $$

where *σ*_*i*,*j*_ is the covariance of biomarker performance between partitions *i* and *j*, *u*_*i*_ is the mean of biomarker performance on partition *i*, and $\sigma _{i}^{2}$ is the variance of biomarker performance on partition *i*.

A confidence interval for the CCC of each set of biomarkers was bootstrapped by re-calculating the CCC using subsets of the available partitions. In particular, we considered all 924 possible subsets of the twelve partitions of size six, and took the range from the 2.5th to the 97.5th percentiles of subset CCC for each biomarker set to derive a 95% confidence interval.

## Results

### Multi-gene biomarkers confer advantage in prognostic performance

**Fig. 2:**
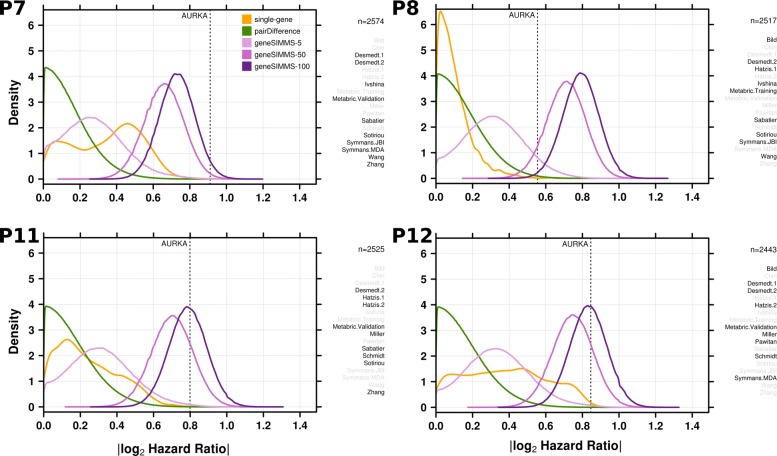
The distribution of biomarker performance on each of the three models tested in four selected meta-dataset partitions. GeneSIMMS biomarkers are separated according to size and the performance of the single-gene AURKA model is displayed. Highlighted datasets comprise the testing cohort in each partition. The total number of patients in the testing cohort is also provided. The plots for the remaining eight partitions can be found in Additional file 1: Figure S1

As shown in four representative partitions (Fig. 2) and in the remaining eight partitions (Additional file 1: Figure S1), we found significant variation in the distribution of single-gene biomarker performance between different partitions of the meta-dataset. That is, even using a minimum of 2282 patients was insufficient to eliminate generalization error. In some cases, the population of single-gene biomarkers had a unimodal distribution of performance, with most genes performing very poorly. In other cases, single-gene biomarker performance was not unimodal and many genes performed well compared to multi-gene biomarkers. But despite these differences, single-gene and pairDifference biomarkers performed worse than multi-gene biomarkers across all partitions. Multi-gene biomarkers also exhibited a much more stable distribution of performance across partitions, with the same unimodal behaviour recurring at roughly the same level of prognostic ability in every meta-dataset partition. This trend was consistent across all signature sizes. Furthermore, larger geneSIMMS biomarkers always outperformed their smaller geneSIMMS counterparts, suggesting some advantage in using more features in a prognostic model.

Although the AURKA single-gene biomarker was within the top percentile of performance distributions among its single-gene peers in each partition, it was consistently outperformed by geneSIMMS biomarkers, especially those of larger sizes (Table 2). The proportion of pairDifference biomarkers offering better prognostic accuracy than AURKA alone was very low. However, thanks to the large number of two-gene models we tested, the total number of models outperforming AURKA was very high: at least 1000 such models in eight out of the twelve partitions and as many as 182,019 models in one partition. By contrast, the number of multi-gene models outperforming AURKA was very large, reaching 7.2% of all 5-gene models in one partition. Indeed the median partition showed ~45% of 100-gene models surpassing AURKA performance. It is thus clear that large numbers of multi-gene models can outperform the best univariate models.

**Table 2:** The proportion of biomarkers outperforming the AURKA single-gene biomarker on each of the twelve meta-dataset partitions by model

Partition	single-gene	pairDifference	geneSIMMS-5	geneSIMMS-50	geneSIMMS-100
P1	3.75×10^−4^	2.25×10^−5^	1.22×10^−3^	0.0289	0.070
P2	7.50×10^−4^	1.75×10^−4^	6.96×10^−3^	0.268	0.570
P3	1.13×10^−3^	9.18×10^−5^	4.60×10^−3^	0.208	0.488
P4	6.50×10^−3^	9.83×10^−4^	0.0178	0.380	0.635
P5	8.75×10^−4^	2.44×10^−5^	6.36×10^−4^	0.129	0.334
P6	3.13×10^−3^	1.43×10^−3^	0.0217	0.407	0.624
P7	7.50×10^−4^	1.31×10^−6^	1.15×10^−4^	7.72×10^−3^	0.0292
P8	1.38×10^−3^	5.69×10^−3^	0.0721	0.925	0.992
P9	1.25×10^−4^	3.72×10^−5^	1.03×10^−3^	0.199	0.483
P10	0	0	3.00×10^−6^	1.23×10^−4^	4.27×10^−4^
P11	6.25×10^−4^	2.11×10^−4^	5.39×10^−3^	0.191	0.451
P12	3.63×10^−3^	6.68×10^−5^	3.97×10^−3^	0.180	0.445

Note that in the case of geneSIMMS, each biomarker size is considered separately

Because most biomarker discovery studies report and recommend a single ‘best’ biomarker for use, we reran AURKA and geneSIMMS-5 with a more restrictive method to directly compare the best reported biomarker from each of the 12 partitions. The |*log*_2_HR| was significantly higher for the best geneSIMMS-5 biomarkers (Paired T-Test; *P*=2.33×10^−12^; mean |*log*_2_HR| = 0.952) than AURKA (mean |*log*_2_HR| = 0.384). This consistent gain in biomarker performance supports the usage of multi-gene methods over the single-gene approach.

### Using multi-gene biomarkers does not compromise performance stability

We observed a large number of multi-gene biomarkers outperforming the AURKA single-gene biomarker in each of the twelve partitions we tested. However, this does not necessarily imply that multi-gene biomarkers are more useful than using AURKA alone for outcome prognosis. The vulnerability of multi-gene models to over-fitting is well-known, which means that the stability of an individual biomarkers’ performance across different meta-dataset partitions is just as important as their performance in any particular partition. As such, we tested the replicability of biomarker performance using the Concordance Correlation Coefficient metric (CCC) and calculated the corresponding bootstrapped confidence interval of stability as described in the Methods section.

**Fig. 3:**
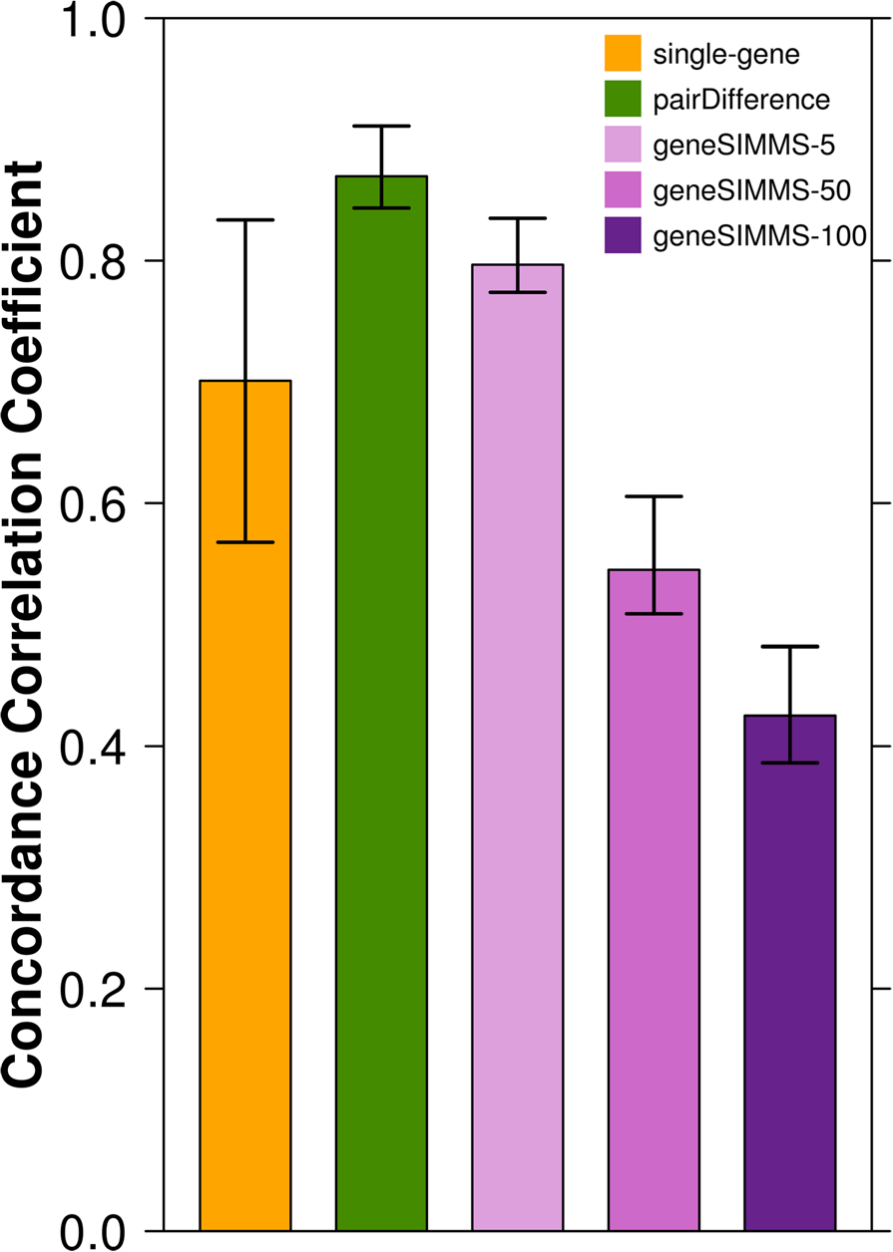
Concordance correlation coefficients of biomarker performance. CCCs from all partitions are displayed by biomarker type (bars). CCCs were re-calculated for all 924 possible subsets of partitions of size six to obtain the 2.5th - 97.5th percentile ranges (whiskers)

Biomarker stability showed clear variation between different models when all partitions were considered as well as when subsets of partitions were tested (Fig. 3). PairDifference biomarkers and geneSIMMS-5 biomarkers exhibited the most consistent performance in different partitions. GeneSIMMS-50 and geneSIMMS-100 biomarkers fared the most poorly in this regard, suggesting there is an optimal size for multi-gene biomarkers. The stability of single-gene biomarkers fell between these two groups. Using a paired Mann-Whitney rank test, we found that the 924 CCCs calculated for each biomarker set were significantly different at the *p*=1×10^−64^ level from that of all other sets. This suggests multi-gene markers, specifically signatures of five genes, can be both more prognostic and more stable than single-gene markers. On the other hand, multi-gene signatures of 50 or 100 genes attain enhanced accuracy at the expense of greater potential to over-fit.

We also considered the biomarkers that outperformed the AURKA single-gene model in all twelve partitions. No such biomarkers were found using the single-gene and pairDifference models. However, one geneSIMMS-5 biomarker, 56 geneSIMMS-50 biomarkers, and 213 geneSIMMS-100 biomarkers satisfied this criterion. Interestingly, only three of these 270 geneSIMMS biomarkers included the AURKA gene, indicating that using combinations of genes that are individually less strongly associated with outcome prognosis can still lead to superior prognostic performance. These 270 biomarkers are listed in Additional file 2.

## Discussion

We comprehensively tested the population of possible biomarkers on twelve unique meta-dataset partitions, which allowed us to observe several intrinsic properties of breast cancer prognostic models. Risk scores calculated by geneSIMMS tended to be much more closely associated with patient outcome than those calculated by models using only one or two genes. However, when larger gene sets were used with geneSIMMS, an over-fitting effect was evident and the performance of individual biomarkers showed less stability across different training and testing patient cohorts. High performance and high stability were found to be optimally balanced in biomarkers of five genes, which outperformed single-gene and gene-pair biomarkers across all metrics. However, it is important to note that the optimal signature size may vary across disease types.

The single-gene hypothesis proposed by Haibe-Kains et al. [16] has been influential having been cited 101 times in the last 10 years. It is therefore critical to re-evaluate important hypotheses like this in the light of technological and analytical advancements. While it is clear that AURKA is one of the best single genes for predicting breast cancer prognosis, it does not necessarily represent the optimal biomarker. To the contrary, many randomly selected gene sets consistently outperform AURKA. This result highlights the critical need for continued development of feature selection algorithms that can maximize this information content. Similarly, our results highlight the importance of considering generalization error carefully when making distributional claims, as any single dataset or partition of training/testing datasets may obscure general trends.

Nevertheless, the single-gene hypothesis may remain valid for other diseases, or even for specific breast cancer subtypes. Multi-gene biomarkers may be better suited to capturing the complex effects of heterogeneous diseases such as breast cancer on mRNA abundance levels, but this may not be true for diseases that only affect a small number of loci or transcriptomic pathways. The judicious comparison of the performance of single-gene and multi-gene biomarkers across many different diseases would greatly aid in the development of clinically useful outcome prognoses from gene expression data.

## Conclusions

Overall, this study highlights the importance of continually re-evaluating older genomic hypotheses in the context of new data and methods (i.e. geneSIMMS). We were able to test our models on a meta-dataset of 4960 patients, over four times the size of the 1,089 patient cohort used by Haibe-Kains et al. [16]. Furthermore, our access to a sizeable compute cluster allowed us to test a large number of all possible gene combinations rather than relying solely on gene sets identified using unproven feature selection algorithms. Future advances in computing and biotechnology will enable even deeper probes and characterization of prognostic biomarker properties.

## Additional files

Additional file 1 **Figure S1:** The distribution of biomarker performance on each of the three models tested in the meta-dataset for partitions not shown in Fig. 2. GeneSIMMS biomarkers are separated according to size and the performance of the single-gene AURKA model is displayed. Highlighted datasets comprise the testing cohort in each partition. The total number of patients in the testing cohort is also provided. (PDF 6349 kb)

Additional file 2 The 270 geneSIMMS biomarkers that outperformed the single-gene AURKA model in all twelve meta-dataset partitions. (TXT 195 kb)

## Abbreviations

CCC: Concordance correlation coefficient
RMA: Robust multi-array analysis
SIMMS: Subnetwork integration for multi-model signatures
