# Supplementary material for: A comparative study of survival models for breast cancer prognostication revisited: the benefits of multi-gene models
Source: BMC Bioinformatics. 2018 Nov 3;19:400. doi: 10.1186/s12859-018-2430-9 (PMC6215649; doi:10.1186/s12859-018-2430-9)
Supplement: Supplementary file 1 — Figure S1: The distribution of biomarker performance on each of the three models tested in the meta-dataset for partitions not shown in Fig. 2. GeneSIMMS biomarkers are separated according to size and the performance of the single-gene AURKA model is displayed. Highlighted datasets comprise the testing cohort in each partition. The total number of patients in the testing cohort is also provided. (PDF 6349 kb) [file 12859_2018_2430_MOESM1_ESM.pdf]

Table S1. A list of the twelve partitions of the breast cancer meta-dataset used in this study, with the proportion of the total number of patients (4,960) used for testing in each partition.

Label	Training Datasets	Testing Datasets	Testing Proportion
**P1**	Chin, Desmedt-1, Desmedt-2, Hatzis-1, Metabric-Training, Pawitan, Sabatier, Symmans-MDA	Bild, Hatzis-2, Ivshina, Metabric-Validation, Miller, Schmidt, Sotiriou, Symmans-JBI, Wang, Zhang	0.527
**P2**	Chin, Desmedt-1, Hatzis-2, Ivshina, Metabric-Training, Pawitan, Schmidt, Sotiriou, Symmans-JBI, Wang	Bild, Desmedt-2, Hatzis-1, Metabric-Validation, Miller, Sabatier, Symmans-MDA, Zhang	0.481
**P3**	Chin, Hatzis-2, Ivshina, Metabric-Training, Pawitan, Sabatier, Schmidt, Wang, Zhang	Bild, Desmedt-1, Desmedt-2, Hatzis-1, Metabric-Validation, Miller, Sotiriou, Symmans-JBI, Symmans-MDA	0.475
**P4**	Bild, Chin, Desmedt-2, Ivshina, Metabric-Training, Miller, Schmidt, Sotiriou, Symmans-MDA, Wang	Desmedt-1, Hatzis-1, Hatzis-2, Metabric-Validation, Pawitan, Sabatier, Symmans-JBI, Zhang	0.466
**P5**	Bild, Desmedt-1, Desmedt-2, Hatzis-1, Metabric-Validation, Miller, Pawitan, Sotiriou, Symmans-JBI, Wang	Chin, Hatzis-2, Ivshina, Metabric-Training, Sabatier, Schmidt, Symmans-MDA, Zhang	0.475
**P6**	Bild, Desmedt-2, Ivshina, Metabric-Training, Miller, Sabatier, Schmidt, Sotiriou, Symmans-MDA, Zhang	Chin, Desmedt-1, Hatzis-1, Hatzis-2, Metabric-Validation, Pawitan, Symmans-JBI, Wang	0.471
**P7**	Bild, Chin, Hatzis-1, Hatzis-2, Metabric-Training, Miller, Pawitan, Schmidt	Desmedt-1, Desmedt-2, Ivshina, Metabric-Validation, Sabatier, Sotiriou, Symmans-JBI, Symmans-MDA, Wang, Zhang	0.519
**P8**	Chin, Desmedt-1, Hatzis-2, Metabric-Validation, Miller, Pawitan, Schmidt, Symmans-MDA, Zhang	Bild, Desmedt-2, Hatzis-1, Ivshina, Metabric-Training, Sabatier, Sotiriou, Symmans-JBI, Wang	0.507
**P9**	Desmedt-1, Hatzis-1, Ivshina, Metabric-Validation, Pawitan, Wang, Zhang	Bild, Chin, Desmedt-2, Hatzis-2, Metabric-Training, Miller, Sabatier, Schmidt, Sotiriou, Symmans-JBI, Symmans-MDA	0.530
**P10**	Bild, Chin, Hatzis-1, Hatzis-2, Metabric-Training, Pawitan, Sabatier, Symmans-MDA	Desmedt-1, Desmedt-2, Ivshina, Metabric-Validation, Miller, Schmidt, Sotiriou, Symmans-JBI, Wang, Zhang	0.517
**P11**	Bild, Chin, Desmedt-1, Ivshina, Metabric-Training, Pawitan, Symmans-JBI, Symmans-MDA, Wang	Desmedt-2, Hatzis-1, Hatzis-2, Metabric-Validation, Miller, Sabatier, Schmidt, Sotiriou, Zhang	0.509
**P12**	Chin, Hatzis-1, Ivshina, Metabric-Training, Sabatier, Sotiriou, Symmans-JBI, Wang, Zhang	Bild, Desmedt-1, Desmedt-2, Hatzis-2, Metabric-Validation, Miller, Pawitan, Schmidt, Symmans-MDA	0.493

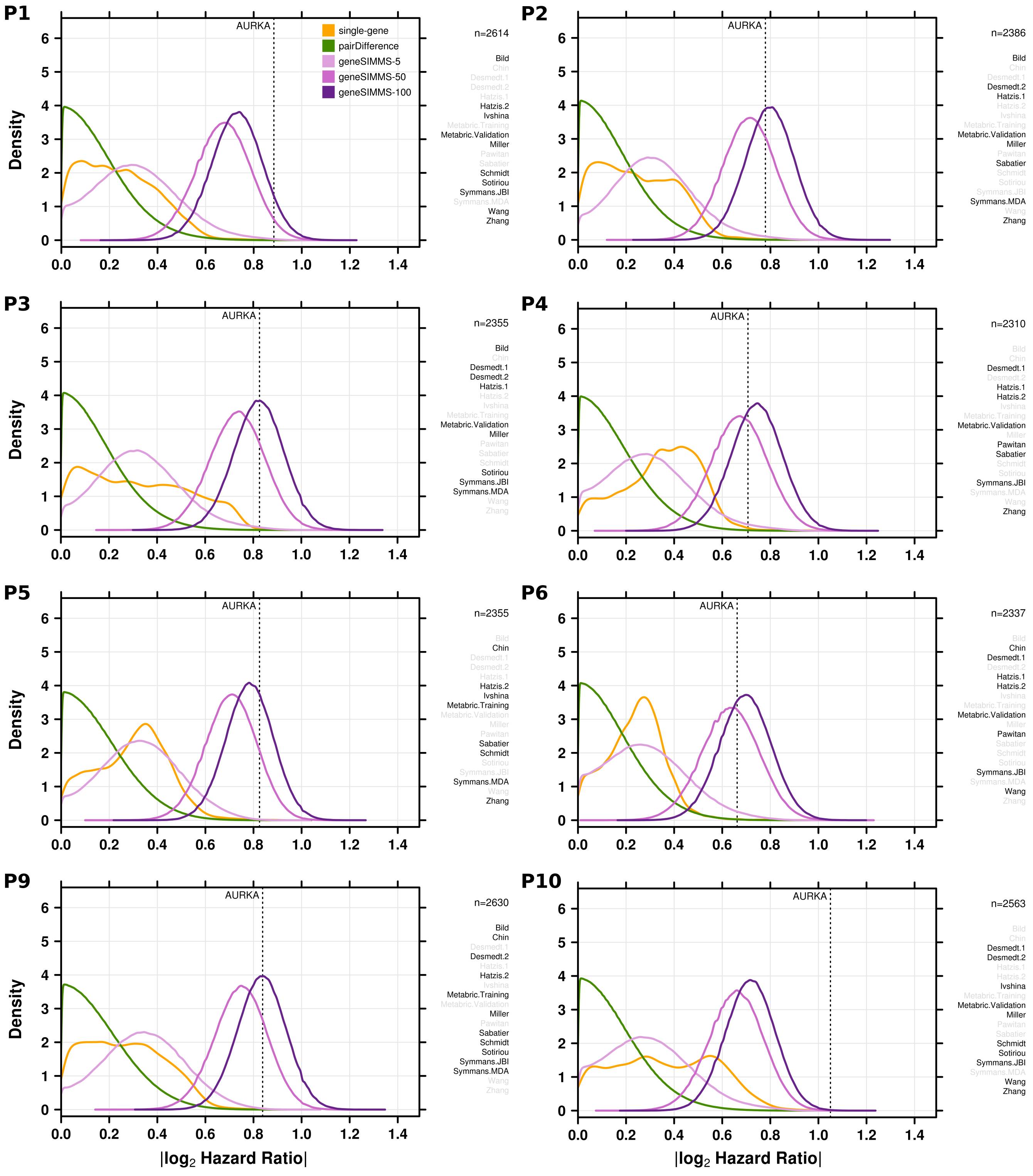

Figure S1. The distribution of biomarker performance on each of the three models tested in the meta-dataset for partitions not shown in Fig. 2. GeneSIMMS biomarkers are separated according to size and the performance of the single-gene AURKA model is highlighted. Highlighted datasets were used for testing in each partition. The total number of patients in the testing cohort is also given.
